# One‐Stage Combined Hip Arthroscopy and Derotational Femoral Osteotomy on a Postless Traction Bed

**DOI:** 10.1002/atn2.70183

**Published:** 2026-07-16

**Authors:** Jack T. Strotbeck, Erel Ben‐Ari, Kyle S. Jamar, Jessica H. Lee, Omer Mei‐Dan

**Affiliations:** ^1^ University of Colorado School of Medicine Aurora Colorado U.S.A.; ^2^ Department of Orthopedics University of Colorado Anschutz Medical Campus Aurora Colorado U.S.A.

## Abstract

Abnormal femoral torsion is associated with labral and chondral tears, hip pain, and early onset arthritis. Derotational femoral osteotomy (DFO) is the surgical treatment of choice to correct femoral torsional deformity, restore normal hip joint mechanics, and improve clinical outcomes. DFO is often performed in conjunction with hip arthroscopy to address intra‐articular pathology, which requires hip joint distraction for visualization. In these cases, the same setup can be carried out seamlessly, allowing for an easy transition between procedures, thus increasing operative efficiency. Traditionally, this combined approach was performed using perineal post traction beds, which carry risks of groin complications and pudendal nerve injury, occasionally resulting in severe consequences. To avoid these complications, postless traction beds were developed to provide safe and reliable distraction without the morbidity associated with perineal posts. This technical note describes the operative management of dual hip arthroscopy and DFO with a postless traction bed offering a safe, reproducible, and efficient surgical technique for managing patients requiring concurrent hip arthroscopy and DFO. Additionally, this paper describes the advantages of the postless traction bed for DFO surgery, even if arthroscopy is not performed concurrently.

## Graphical abstract

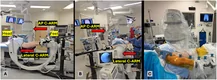

**VIDEO 1:** Video for left sided derotational femoral osteotomy. Patient positioned supine on a postless traction table for hip arthroscopy. Video content can be viewed at https://doi.org/10.1002/atn2.70183.

Anatomic femoral antetorsion ranges from 15° to 20° with lesser values defined as retrotorsion whereas greater values indicate excessive antetorsion.^1^ Abnormal femoral torsion increases joint stress and can lead to labral and chondral damage, hip pain, and early onset arthritis.^1^, ^2^, ^3^, ^4^, ^5^, ^6^ This condition may arise in isolation or in association with femoral acetabular impingement or dysplasia, contributing to hip instability that may require hip arthroscopy and/or periacetabular osteotomy in addition to derotational femoral osteotomy (DFO).^7^, ^8^, ^9^

Prompt recognition and correction of pathologic torsion via DFO can yield excellent outcomes.^10^ Restoration of normal torsional alignment improves joint mechanics, reduces pathological joint stress, and may prevent cartilage damage and the need for total hip arthroplasty.^10^, ^11^, ^12^, ^13^, ^14^, ^15^, ^16^

DFO is typically performed by osteotomizing the femur and rotating the distal fragment to restore limb alignment.^17^ Precise traction and controlled manipulation of the distal fragment are essential to achieve accurate correction. Historically, DFO was performed using free‐leg positioning or peroneal post traction beds, both of which present technical challenges. Free‐leg setups limit distal fragment control, whereas perineal posts may impair imaging and increase risk of groin injuries, perineal injuries, and other reported disadvantages.^16^, ^18^, ^19^

The use of postless traction beds in hip arthroscopy and single‐stage arthroscopy with periacetabular osteotomy have been previously published.^9^, ^20^ This paper details the specific surgical technique using the postless Pivot Guardian Distraction system (Stryker, Kalamazoo, Michigan) for combined hip arthroscopy and DFO, as well as for isolated DFO, and highlights its advantages over other techniques using tractionless or traditional perineal post traction beds.

## SURGICAL TECHNIQUE

**FIGURE 1:**
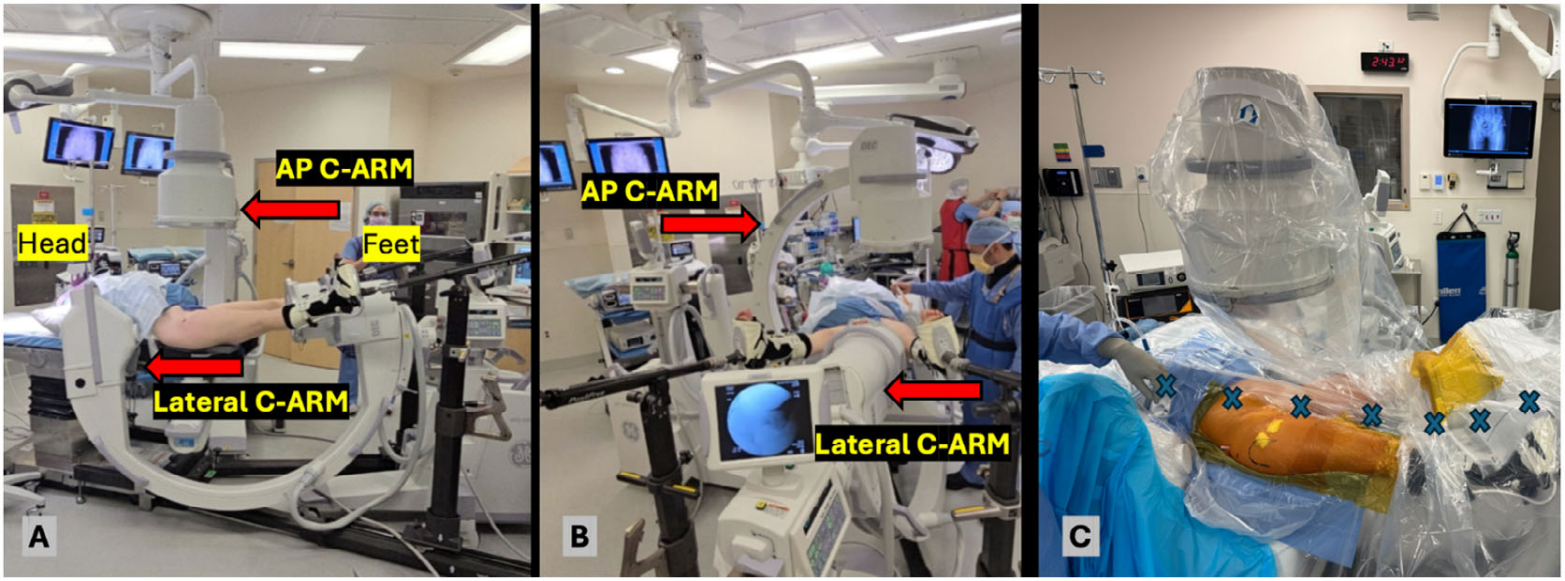
Patient positioning and draping for one‐stage combined hip arthroscopy and DFO of the right hip using a postless traction setup. The patient is supine with their feet in the traction boots. (A,B) Pre draping setup where red arrows indicate the dual AP (top arrow) and lateral (bottom arrow) C‐arms. (C) Single drape setup for both arthroscopy and DFO (Blue “X”s) starting proximal to the iliac crest extending to the knee joint using postless Guardian Traction Table. (AP, anteroposterior; DFO, derotational femoral osteotomy.)

The patient is positioned supine on a postless distraction table (Pivot Guardian, Stryker, Kalamazoo, Michigan) regardless of whether concomitant hip arthroscopy is planned. Unlike traditional perineal post traction beds, the postless bed eliminates postrelated positioning challenges and enables full bilateral leg access, optimizing both surgical field preparation and intraoperative fluoroscopic imaging. After hip arthroscopy is completed, the position is maintained, with the nonoperative leg abducted either straight or slightly flexed to allow space between the legs for the C‐arm lateral imaging. A single prep draping layout is used for both procedures with draping beginning proximal to the iliac crest and extending to the knee joint. This setup allows for a seamless transition between arthroscopy and DFO. For combined arthroscopy and DFO cases, hip arthroscopy is performed first as previously described^10^ and once completed skin portals are closed. The C‐arm units are then positioned for the subsequent DFO. The C‐arm used for arthroscopy is maintained between the patient's legs to obtain the lateral view. The second C‐arm is brought in from the nonoperative side for the anteroposterior view (Figure 1). The postless bed radiolucent extension, combined with simultaneous anteroposterior and lateral imaging, allows confirmation of nail position and assessment of osteotomy alignment in multiple views. This approach reduces the time required to obtain satisfactory intraoperative radiographs, thereby decreasing overall operative time, blood loss, and potential infectious exposure.^9^

**FIGURE 2:**
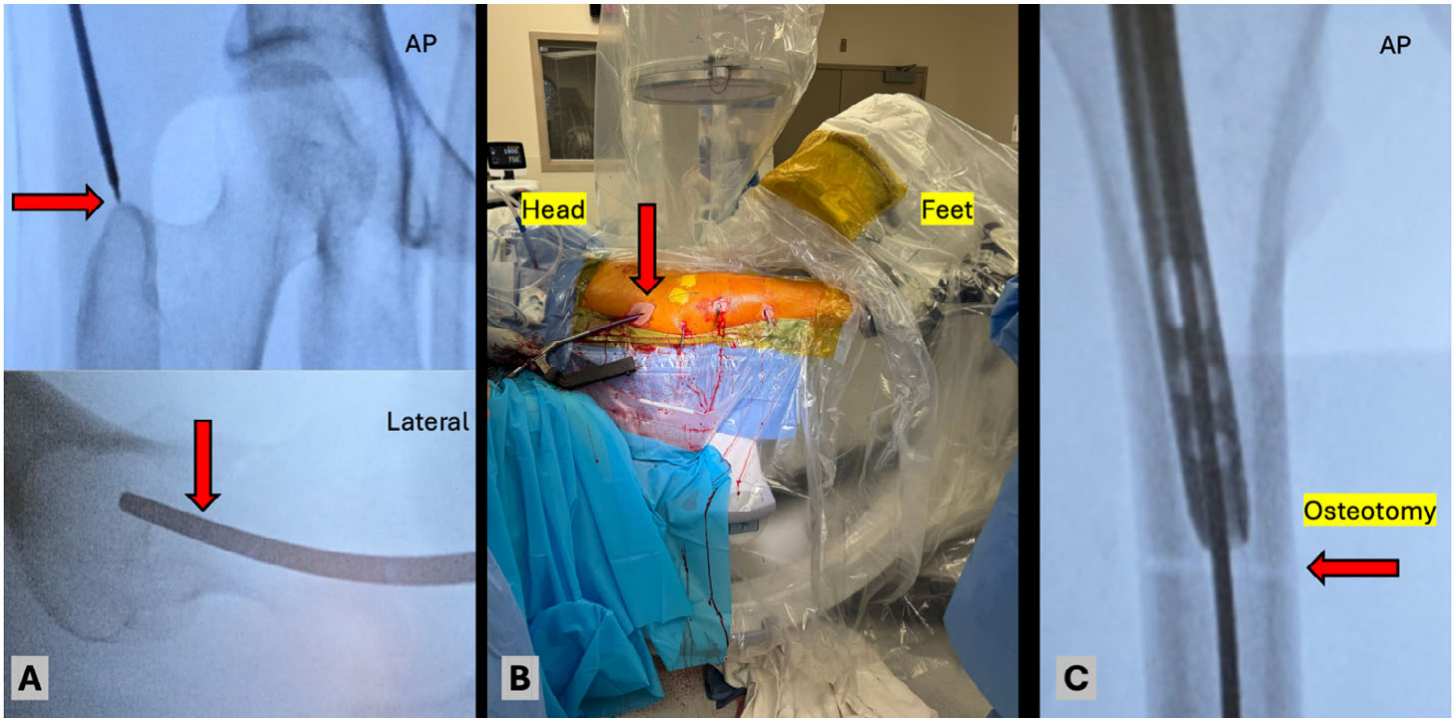
Sequence prior to osteotomy for DFO of the right hip. The patient is supine with their feet in the traction boots. (A) Simultaneous acquisition of AP and lateral radiographic views of the right femur showing guide wire (red arrow) insertion at the tip of the greater trochanter. (B) Side view of a right femoral nail inserted from proximal and advanced distally. (C) AP radiographic view of a right femoral nail advanced just proximal to the osteotomy site. (AP, anteroposterior; DFO, derotational femoral osteotomy.)

A 4 cm incision is made proximal to the greater trochanter. An intramedullary nail (InterTAN, Smith & Nephew, Memphis, TN) entry point at the tip of the greater trochanter is established with a drill guide under fluoroscopic guidance (Figure 2). This step is followed by entry reamer and introduction of a ball‐tip guidewire into the femoral canal. A measurement is obtained for optimal nail length. Prior to canal reaming, the planned osteotomy site is confirmed under fluoroscopy and marked with a 2.3 mm drill guide (InterTAN, Smith & Nephew, Memphis, TN), which also vents the canal to reduce the risk of fat embolism during reaming.

**FIGURE 3:**
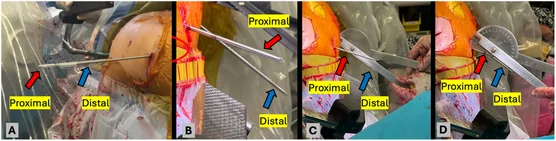
Schanz pin alignment throughout torsional correction during right DFO. Red arrow pointing at proximal pin and blue arrow pointing at distal pin. (A) Distal‐to‐proximal view: Pre torsional correction parallel pin positioning. (B) Proximal to distal view: Post torsional correction pin alignment. (C) Proximal to distal view: Intraoperative measurement of femoral torsional correction with a goniometer using the distal Schanz pin as reference. (D) Proximal to distal view: Intraoperative measurement of femoral torsional correction with a goniometer using the proximal Schanz pin as reference. (DFO, derotational femoral osteotomy.)

With the leg stabilized by the traction device, the femoral canal is reamed in 0.5 mm increments to the level of the planned position of the nail tip. Nail width is determined based on reaming chatter, and reaming is carried out 1 to 1.5 mm beyond the determined nail width to facilitate nail insertion. Following canal preparation, a 14 mm entry reamer is advanced to the level of the lesser trochanter to match the proximal nail width, and the nail is inserted to just above the osteotomy site. Two 4 mm Schanz pins are drilled into the femur in parallel to mark the preosteotomy alignment and establish a baseline angle to guide torsional correction (Figure 3). The proximal pin is positioned at the level of the greater trochanter, posterior to the nail, with enough clearance to advance the nail without abutting the pin, and a second pin is positioned at the distal femur, midway between the isthmus and the nail tip. It is important to make sure both pins are bicortical. A 2.3 mm drill (InterTAN, Smith & Nephew, Memphis, TN) is then used to create several drill holes in a sun‐ray pattern at the planned osteotomy site to circumferentially perforate the femoral cortex, facilitating a controlled osteotomy and minimizing the risk of fracture propagation. The guidewire is withdrawn to just proximal to the osteotomy, which is then completed using a ¼inch osteotome. Once the osteotomy is complete, the guide wire is inserted to the distal femur and slight traction is applied to disengage and better align the 2 femoral fragments. This level of traction (30‐50 Lbs) relies on flat body friction against the postless bed and does not require Trendelenburg position or a perineal post, thereby eliminating the risk of perineal compression and groin injuries.^20^

**FIGURE 4:**
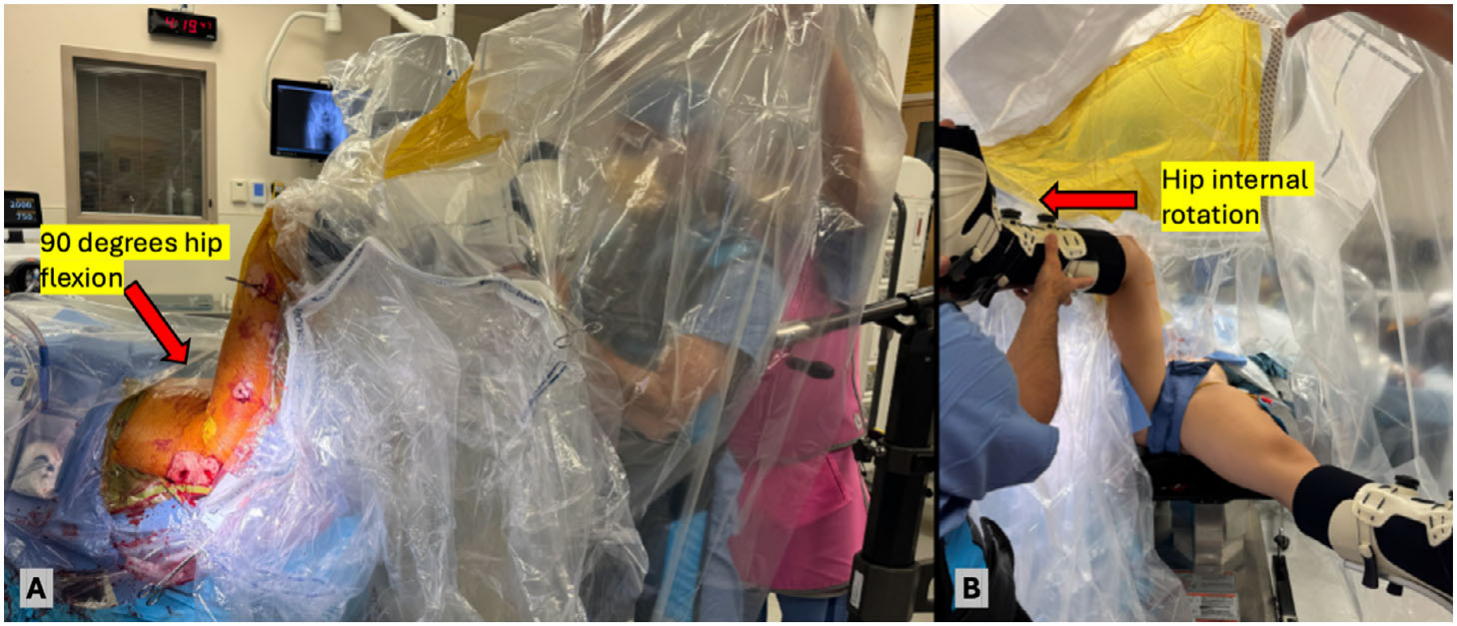
Right hip range‐of‐motion evaluation following DFO torsional correction (A) Side view: showing how the hip is brought to 90° of flexion and (B) Caudal to cephalad view from under the drapes showing hip internal rotation range‐of‐motion evaluation after correction. (DFO, derotational femoral osteotomy.)

The distal femoral fragment can then easily be manipulated in all planes using the traction device as an unscrubbed assistant moves the boot internally or externally per the required correction, while the scrubbed assistant holds onto the nail handle proximally to stabilize it, and the surgeon moves the distal pin alongside the foot motion. The angle of torsional correction between the Schanz pins is measured using a goniometer (Figure 3). Additionally, comparison of boot orientation before and after correction provides a secondary visual reference to confirm femoral torsional correction. Once correction angle is confirmed, the nail is advanced distal to the osteotomy site over the ball‐tip guidewire. As the nail passes the osteotomy site and the distal femur stabilizes, rotational alignment is rechecked with the goniometer, as nail entry into the distal fragment may cause subtle changes in alignment and torsional correction. Adjustments to obtain optimal correction can still be made at this point. If correction is deemed optimal and stable after further advancement of the nail, the nail is advanced to its final position and locked proximally with an intertrochanteric screw. Next, a lateral radiograph is used to apply the “perfect circle” technique^21^ for positioning the drill guides into the distal locking holes, utilizing the oblong and 15° oblique holes to achieve interim divergent stabilization of rotational motion, which is critical during range‐of‐motion assessments. After removing the nail handle, the boot is disengaged from the traction bed, and the hip is flexed to 90° to measure postcorrection internal rotation as a means of verifying desired torsional correction (Figure 4).^22^ If hip range‐of‐motion aligns with the planned correction, the boot is reconnected to the bed, and the drill guides are replaced with distal locking screws. If slight over‐ or under‐correction is observed, fine tuning can still be performed: the drill guides are removed, the proximal nail is restabilized using the nail handle or a screwdriver at the proximal locking screw, and the distal fragment is rotated around the nail using unicortical Schanz pins to achieve the desired alignment.

## DISCUSSION

DFO, the surgical solution for femoral malrotation, was historically performed with free‐leg positioning or a perineal post traction bed.

This technique differs from previous methods, allowing seamless transition from hip arthroscopy to DFO using a postless traction bed. It provides excellent control of the femur before and after osteotomy, facilitating distal fragment manipulation, precise incremental adjustments, and stable fixation, while avoiding the limitations associated with free leg and post traction beds techniques. The postless distraction system allows efficient positioning and simultaneous use of the C‐arm to obtain anteroposterior and lateral views without readjustment. This approach represents a substantial improvement over traditional techniques, and may reduce operative time, lower infection risk, and enhance overall operative efficiency.

Several authors have described DFO without traction assistance.^16^, ^18^, ^19^, ^23^, ^24^, ^25^ However, these techniques have notable drawbacks. Many are free‐leg, limiting distal femoral fragment control, causing difficulties with surgical stability, torsional correction, fixation, and the need for redraping when DFO is performed in conjunction with hip arthroscopy.^16^, ^18^, ^19^, ^23^ Others describe techniques that involve a seamless setup for hip arthroscopy and DFO using a traditional perineal post traction device.^26^ Although it offers improved operative efficiency and the advantages of traction control for the DFO, it exposes patients to unnecessary risks such as groin injury, pudendal nerve palsy, and genital soft‐tissue damage.^27^, ^28^, ^29^ The post additionally interferes with obtaining satisfactory radiographs during the surgery by adding metal artifact and requires multiple image acquisitions with single C‐arm repositioning. Additionally, the post places a force on the proximal fragment, displacing it into abduction, potentially complicating reduction efforts.

Potential drawbacks to this technique should be noted. First is the availability of the postless traction table. Although postless traction tables are a far safer alternative to traditional perineal post traction, they may not be available in all operating rooms. Another potential risk to using traction beds for a DFO is traction‐related injury to the operative leg. However, the traction used in DFO is far below what is used during hip arthroscopy, and there is no evidence of this complication with a postless traction bed either in the published literature or from our clinical experience^30^ (Table 1).

**TABLE 1:** Key Advantages and Disadvantages of Postless Arthroscopy Traction Table

ADVANTAGES
• Allows hip distraction without traction against post
• Short learning curve transitioning from perineal post traction to postless traction tables
• Ability to address pathologic femoral torsion and intra‐articular pathology with DFO and hip arthroscopy in a single setup
• Easy transition from arthroscopy to DFO with same operative set up without need to transfer patient or re‐drape which may reduce infectious exposure, bleeding, and time under anesthesia
• Traction force can be measured and controlled when disengaging the osteotomized femur
• Allows for simultaneous anteriorposterior and lateral fluoroscopic views during surgery
• Easy leg manipulation in all directions (up/down, foot/femur rotation, abduction/adduction)
• Easy foot release mechanism enables post torsional correction range‐of‐motion evaluation
• Easy traction set up similar to fracture table
• Can be used for fixation of traumatic femur fractures
• Radiolucent extension table accommodates patients over 6 feet in height

DISADVANTAGES
• Postless traction tables may not be accessible to all facilities and surgeons as part of a routine operating set up

DFO, derotational femoral osteotomy.

In conclusion, postless traction using the Pivot Guardian represents a significant technical advancement for single‐stage hip arthroscopy and DFO. Most notably, it provides seamless transition and controlled traction for both procedures in the same setup. Additionally, the elimination of perineal post traction related complications, easy workflow integration, and superior imaging acquisition support implementation of this technique for hip arthroscopy and DFO surgery. Pearls and pitfalls are shown in Table 2 and the technique is illustrated in Video 1.

**TABLE 2:** Derotational Femoral Osteotomy‐Pearls and Pitfalls

Pearls	Pitfalls
• Use a 2.3 mm drill to create circumferential perforations along the planned osteotomy site prior to osteotomy use to minimize the risk of unintended fracture propagation	• Avoid nail entry point too far posterior to facilitate proper placement of the proximal Schanz pin posterior to the nail
• Advance the guidewire distally immediately after completing the osteotomy. This prevents translation of the femoral fragments which complicates wire passage	• After completing torsional correction, advance the nail to its final position gradually and in a controlled, stepwise manner rather than continuously, and keep on measuring torsional correction as nail advancement may inadvertently alter the achieved rotational alignment as nail abuts cortical bone
• Ensure that the nail handle and proximal Schanz pin are securely stabilized during torsional correction to maintain rotational control	• Avoid excessive traction after the completion of osteotomy. Release traction once rotational alignment is achieved to prevent fracture gapping and loss of correction
• Use the oblong and 15° circular holes for distal locking screws placement to control femoral rotation during intraoperative range‐of‐motion assessment and enhance overall construct stability	• Avoid unicortical placement of Schantz pins by using anteriorposterior and lateral views during entry of pins
• Allow for weightbearing as tolerated immediately postoperatively	

## DISCLOSURES

The authors (J.T.S., E.B‐A., K.S.J., J.H.L., O.M‐D.) declare that they have no known competing financial interests or personal relationships that could have appeared to influence the work reported in this paper.
